# Intraoral medical devices for sustained drug delivery

**DOI:** 10.1007/s00784-023-05377-5

**Published:** 2023-11-20

**Authors:** Suhail Alghanem, Ewelina Dziurkowska, Iwona Ordyniec-Kwaśnica, Małgorzata Sznitowska

**Affiliations:** 1 Department of Pharmaceutical Technology, Faculty of Pharmacy, Medical University of Gdansk, Al. Gen. J. Hallera 107, 80-416 Gdansk, Poland; 2 Department of Analytical Chemistry, Faculty of Pharmacy, Medical University of Gdansk, Al. Gen. J. Hallera 107, 80-416 Gdansk, Poland; 3 Department of Dental Prosthetics, Faculty of Medicine, Medical University of Gdansk, Str. E. Orzeszkowej 18, 80-208 Gdansk, Poland

**Keywords:** Oral cavity, Medical device, 3D printing, Microneedle patch, Iontophoresis

## Abstract

**Objectives:**

The oral cavity constitutes an attractive organ for the local and systemic application of drug substances. Oromucosal tablets, gels, or sprays are examples of the formulations applied. Due to the elution through the saliva, the residence time of the formulation at the application site is relatively short. Medical devices placed in the oral cavity, with a reservoir for an active substance, play an important role in solving this problem.

**Materials and methods:**

In this review, we discuss the devices described in the literature that are designed to be used in the oral cavity, highlighting the advantages, disadvantages, and clinical applications of each of them.

**Results:**

Among the intraoral medical devices, special types are personalized 3D-printed devices, iontophoretic devices, and microneedle patches.

**Conclusion:**

We anticipate that with the development of 3D printing and new polymers, the technology of flexible and comfortable devices for prolonged drug delivery in the oral cavity will develop intensively.

**Clinical relevance:**

The presented review is therefore a useful summary of the current technological state, when in fact none of the existing devices has been widely accepted clinically.

## Introduction

Among different drug delivery routes, oral drug delivery is by far the most accepted and trusted mode of drug administration, mainly owing to its patient compliance, ease of application, and reasonable pricing [13]. Solid oral dosage forms (tablets, pills, and capsules) are excellent drug delivery systems, but only if patients can swallow them [46]. Many pharmaceutical dosage forms are applied in the oral cavity; some of them are solid preparations (buccal tablets, disintegrating tablets); others are semi-solids (gels, ointments) or solutions (sprays, mouthwashes) [62].

Drug delivery via the oral mucosa can be subdivided into three different approaches: sublingual delivery across the mucosa lining the floor of the mouth, buccal delivery via the mucosa lining the cheeks (both for the systemic delivery of drugs), and not site-specific delivery into the oral cavity for the treatment of oral conditions, i.e., the local delivery of drugs [15]. Local drug delivery allows topical treatment of various oral mucosal diseases, as it provides a more targeted and efficient drug delivery option than systemic delivery in this case. The oral cavity constitutes an attractive organ for the systemic application of drug substances due to its anatomic composition and high vascularization. For some drugs, this route of drug delivery, providing direct entry into the systemic circulation, allows to avoid the first-pass effect [4, 68].

Currently, the interest in oral mucosal permeability is related to the opportunity to achieve prolonged and controlled drug delivery [69]. Residence time in contact with the oral mucosa of disintegrating tablets, buccal tablets, lozenges, chewing gums, sprays, mouthwashes, breath fresheners, and gels is limited due to the physiological removal mechanisms of the oral cavity: the washing effect of saliva and mechanical stress. These dosage forms are constantly washed away, resulting in retention times of insufficient duration, unpredictable distribution of the drug on the site of action/absorption, and an initial burst effect, followed by a rapid decrease in concentrations to below therapeutic levels [52, 62]. Residence time for most mucosal routes is less than an hour, typically in minutes and the saliva washes out the drug substances into the stomach. Mucoadhesive agents allow to localize the delivery system and increase the contact time at the site of absorption for hours [81]. Mucoadhesive systems for oral local drug delivery include adhesive tablets, films, gels, ointments, or sprays. The most prolonged adhesion has been reported for oral films and patches [74, 80]. However, this was rarely more than a few hours. Moreover, in the case of oral mucoadhesive solid dosage forms, involuntarily swallowing or choking is a serious risk [6].

**Fig. 1:**
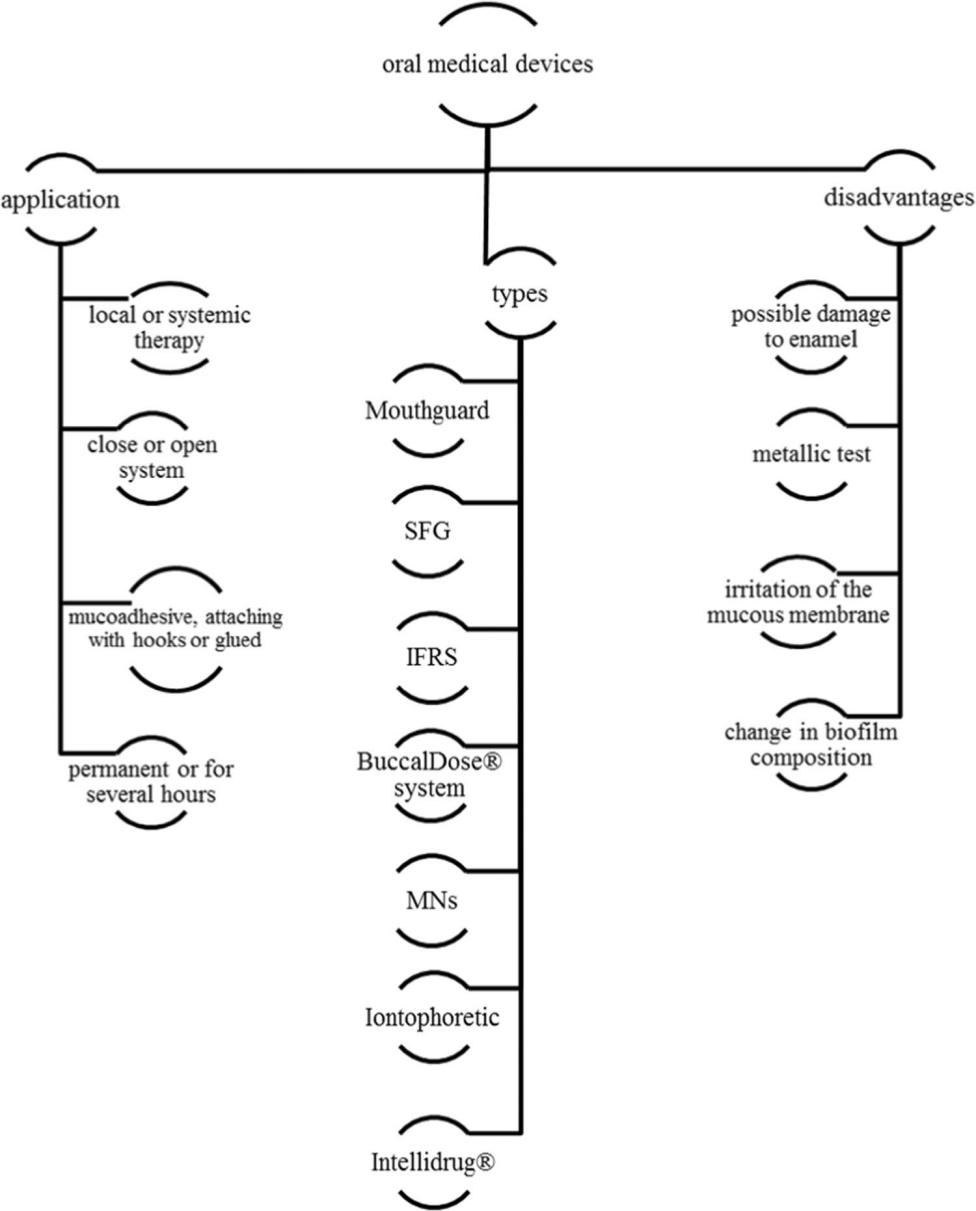
Graphic presentation of the issues discussed in the manuscript

All these challenges encourage scientists, engineers, and physicians to create new strategies for drug delivery from the oral cavity, including the concept of using medical devices as drug reservoirs, sometimes with the function of controlling the drug release rate. This is an attractive option nowadays because using 3D printing techniques, it is easier to design a personalized device that fits the patient [41, 52]. The aim of the paper is to review the current literature describing such devices and their functionality. The issues raised in the manuscript are illustrated in Fig. 1.

## The anatomy and physiology of the oral cavity

The oral cavity consists of an outer vestibule and an inner oral cavity proper. The oral cavity comprises various structures containing gum (gingiva), teeth, soft and hard palate, tongue, mucosal membrane of the cheek’s inner surface, lips, and supporting tissues [32]. In addition, there are many nerve endings responsible for the sense of taste [60].

The mucous membrane is composed of two layers: the surface epithelium and an underlying connective tissue layer, the lamina propria. As a result of the variability in the type of epithelium present, as well as the characteristics of the connective tissue, several regions can be distinguished: lining mucosa, masticatory mucosa (mucoperiosteum), specialized mucosa, and a transitional zone (vermillion zone) [8]. The oral mucosa has a total surface area of about 200 cm^2^ [62] but the total surface area available for drug absorption is 170 cm^2^ of which about 50 cm^2^ represents non-keratinized tissues: while lining (buccal) mucosa is composed of lightly keratinized epithelium, masticatory mucosa (gingiva) is highly keratinized. The dorsum of the tongue is covered by a specialized epithelium, which can be represented as a mosaic of keratinized and non-keratinized epithelium [9, 19, 22, 78].

The cells of the oral epithelia are surrounded by an intercellular ground substance, mucus, that has a structure of hydrogel of approximately 83% water containing diverse compounds, with mucin as the main component, mainly produced by goblet cells of the epithelium. Mucin is represented by glycoproteins of diverse molecular weights ranging between 1 and 40 × 10^6^ Da and defines the 3D network of the mucus determining its viscous properties [71, 81].

The oral cavity is also characterized by high humidity, guaranteed by the presence of saliva and crevicular liquid [27]. Human major salivary glands consist of three pairs of glands known as parotid, submandibular, and sublingual glands; together, they are responsible for about 90% of the saliva. The salivary glands play an important role in maintaining a moist surface containing mucins and a variety of antimicrobial substances as well as epidermal growth factor (EGF) [78]. Generally, salivary glands produce on average between 800 and 1500-ml saliva each day, which would correspond to the saliva production rate of approximately 700–1000 μl/min [69].

The main components of saliva include water 99%, mucus, mineral salts, and proteins including enzymes (amylase, lysozyme, and lipase). The normal pH of saliva is 6 to 7, with a range from 5.3 (low flow) to 7.8 (peak flow) [11, 85].

## Medical devices

Medical devices which are used to increase the residence time of the drug in the oral cavity contain the drug reservoir, the element locating the device in the oral cavity, and optionally the system controlling the drug release rate. They can be for single or multiple uses. The drug can be released either to the saliva (“open system”) or unidirectionally through the oral mucosa to the blood (“closed system,” which protects the active ingredient of the elution by saliva). The open system may be used for both local therapy (e.g., antibiotic) and systemic therapy (e.g., naltrexone).

The systems used differ not only in design but also in the mode of application, principles of the release of active substance, and indications for topical or systemic delivery, as presented in Table 1.

**Table 1:** Types of devices used for drug delivery in the oral cavity

Medical device	Mode of application	Drug substance	Drug release and delivery	References
Mouthguard 3D printed	Removable device/for temporary use	Clobetasol propionate, chlorhexidine, and sodium fluoride	Diffusion in the polymeric matrix forming the device, intraoral delivery	[3, 7, 12, 37, 49]
Slow-release fluoride glass (SFG) pellets	Single use/temporary	Sodium fluoride	Slow dissolution of the gellifying matrix, intraoral delivery	[20, 83]
Intraoral fluoride-releasing system (IFRS)	Fixed device/temporary	Sodium fluoride	Diffusion from a polymeric matrix through a rate-controlling membrane; intraoral delivery	[17, 18, 58]
BuccalDose® system	Fixed device/permanent, with disposable cartridge	No drug substance was used; the device is under preclinical development	Osmotic pump delivering the drug through a membrane; the drug delivery is directed towards the buccal mucosa	[33, 34, 79]
Microneedles (MNs)	Removable device/temporary	Sulforhodamine, doxorubicin, ovalbumin, HIV antigens, cholera toxin, human insulin, human growth hormone	Following “injection,” the coating layer containing the incorporated active substance is slowly dissolving and the drug is absorbed in the mucosal tissue and penetrates to the blood	[14, 53, 54, 58, 61, 77]
Iontophoretic devices EAER®, Fluorinex®	Electrode placed in the oral cavity while the whole device is located externally	Acidulated phosphate fluoride, sodium fluoride, atenolol HCl, lignocaine + epinephrine, buflomedil HCl + dexamethasone sodium phosphate, lidocaine + nicotine, fluoride	Transmucosal absorption of a drug solution by an application of an externally applied current potential	[30, 56, 66, 67]
Iontophoretic patch	Removable device/temporary	Prilocaine and lidocaine HCl	Transmucosal absorption of a drug from a polymeric patch consists of 3 layers: release layer, mucoadhesive layer and packing layer	[24]
IntelliDrug®	Fixed device, with a reservoir to refill	Naltrexone, galantamine, levodopa	Transmucosal delivery of a drug solution from a reservoir through a membrane, by an osmotic mechanism and application of an externally applied current potential	[16, 28, 63, 75]
Combination of MNs and iontophoresis	Removable device/temporary	Lidocaine HCl	Microporation of the buccal membrane followed by application of a gel	[76]

### Mode of application

The device is usually placed by attaching it to the teeth with hooks (intraoral fluoride-resealing system (IFRS)). Smaller systems may be mucoadhesive—they adhere to the gum or cheek or may be glued to the tooth (slow-release fluoride glass (SFG) pellets). 3D printing makes it possible to personalize the construction and to form the shape fitting to the individual toothing that holds the system on the teeth or between teeth when there are missing (teeth bridges) (BuccalDose® system and IntelliDrug®). The systems are most often designed to remain in the mouth for at least several hours and therefore should be comfortable, but also safe—they cannot pose a risk of falling off and choking. Some systems do not allow their use during meals (mouthguard) and are very often intended to be placed in the mouth at night. Thus, for hygiene reasons or if during meals the removal is required, the patient should be able to install the system on his own. Permanent systems with replaceable inserts are also known, e.g., IntelliDrug®.

The main problem with the use of such devices concerns patient’s comfort and compliance. It is most important that the application does not cause ulcers and inflammations and other problems concerning microbiological safety or the method to refill the reservoir. Other challenges include food intake, mechanical forces, and saliva flow. To date, a limited number of studies have been carried out on the applications of these devices in vivo.

### Principles of the release mechanism of the active substance

The medical device contains a reservoir for the active substance in the form of a solution, a gel, or a slowly dissolving gelling matrix. It is closed with either a semi-permeable or a perforated membrane. The release of the drug occurs as a result of diffusion caused by the concentration gradient—in the reservoir and the saliva (or in the mucous membrane), supported sometimes by the erosion of the reservoir/matrix. The penetration of saliva into the reservoir and dissolution of the drug substance, which may be in solid form, are also important. 3D printing technology made it possible to incorporate the active substance into the device material, shaped as a tooth cap (e.g., mouthguard). In this way, the entire system is loaded with a drug, serving as a reservoir, and the release occurs due to diffusion in the polymer building the device and sometimes due to surface erosion of the polymer.

As already mentioned, there are possible two release routes of the drug substance from the device—into saliva (“open system”) or into the mucous membrane with which the reservoir is in contact (transmucosal absorption). In the latter case, the reservoir is most often closed from the side of the oral cavity, which prevents the active substance from being released into the saliva. After absorption through the mucosa, the drug is supposed to reach the bloodstream and exert a systemic effect. Table 1 presents different medical devices with characteristics of the mechanism of the drug release mode.

Each mucosal area in the oral cavity has different barrier properties and permeabilities. The knowledge of barrier properties and mechanisms of membrane transport is essential for drug delivery technologies [86]. Keratinized mucosa (e.g., buccal or gingival region) is several times less permeable than non-keratinized mucosa (e.g., sublingual region) which means that the drug absorption is small and it is a challenge to achieve therapeutic blood level of the active substance when systemic action is required. Larger molecules like peptides do not penetrate effectively even through non-keratinized mucosa. As described further, iontophoresis is the most popular physical method that improves drug absorption through the barrier. The epithelium of the mucosal membrane is built by 40–50 layers of the cells, and drugs are absorbed through the mucosa through either the transcellular or paracellular route. Once the drug has overcome the mucus and epithelium barrier, it can be absorbed via the venous drainage to the internal jugular vein and enter the systemic circulation directly, bypassing first-pass metabolism and gastrointestinal drug degradation. The buccal route can be useful for extended drug release by increasing the mucosal adhesion time while it can still achieve a relatively rapid onset of action. If the active substance is released intraorally, i.e., into saliva and not directly into the mucous membrane, its transmucosal systemic absorption is limited, but it can be used for local treatment of diseases of the oral cavity or throat [44, 45].

### Safety and comfort of the device

When administering a drug to the oral cavity, the chemical and mechanical properties of the medicine or the system used should be taken into account. In the first case, the limitations result primarily from the pH of the preparation, taste, irritating potential, or toxicity of the active substance. Low pH, although inhibiting the growth of microorganisms, with long-term contact may destroy of tooth enamel. The cause of the unacceptable taste may be not only the active substance but also the components of the device placed in the mouth which may induce metallic taste.

The mechanical forces exerted by the installed medical device should be taken into account as an important factor determining the patient’s comfort, and mechanical damage to the mucous membrane will be particularly dangerous. This effect is related to the type of tissue under the mucosa: the connective tissue of lining mucosae is more elastic and flexible than the connective tissue in the gingiva [78]. Any change in the mechanical properties of the mucous membrane makes it more vulnerable to injuries and damage and reduces the pressure pain threshold. Most of the pain experienced when wearing a removable denture is due to damage to the underlying tissue due to overloading [19]. On the other hand, the device installed in the oral cavity is subjected to a harsh environment including mechanical forces of (up to 250 N) [31, 70].

The oral cavity is a complex ecological niche as more than 700 microorganism species colonize the oral cavity, which is closely associated with oral health [50]. Drugs and devices applied are among many chemical and physical factors that influence the oral microbiota. Medical devices may constitute an additional substrate for the development of biofilm and therefore regular hygiene procedures are needed, so it is advantageous to design devices that can be removed for cleaning [27].

Regarding mouthguards, polymeric materials used in their fabrication should be elastic, disinfectable, easy to clean, tasteless, and odorless [39]. However, decontamination procedures may cause damage to the device surface causing deterioration of their properties and favoring pathogen colonization [55]. Other studies indicated that steel-based elements (dental crown attachments and orthodontic brackets) degrade due to surface pitting and inherent cracks being consequences of biocorrosion and mechanical stresses, also during cleaning, which can lead to bad metallic taste in patients who placed these devices for a long time [25].

### Types of medical devices

In the following sections, we will review the literature reporting the construction and the *in vitro* and the *in* vivo studies conducted to evaluate the performance of different types of medical devices (Table 1). Special types of the devices are patches with microneedles and those based on iontophoretic transport, as in these cases, the promotion of transmucosal penetration is achieved by changing the permeability of the buccal mucosa or dental hard tissue.

Among the active substances studied are most often agents for Parkinson’s disease or dry mouth, antiseptics, anti-inflammatory, analgesics, or fluoride supplementation. Besides the medical devices for drug delivery, also, devices modifying the environment or physiology of the oral cavity in a non-pharmacological way are described in the literature but they are in the scope of this review. Worth mentioning are, for example, electrostimulators like Salitron®, GenNarino®, and SaliPen® that stimulate saliva secretion in patients suffering from dry mouth [26, 72]. They have been developed as drug-free systems stimulating a lingual nerve but it may be the idea that can be also used in the future in combination with pharmacological treatment.

### Mouthguards

**Fig. 2:**
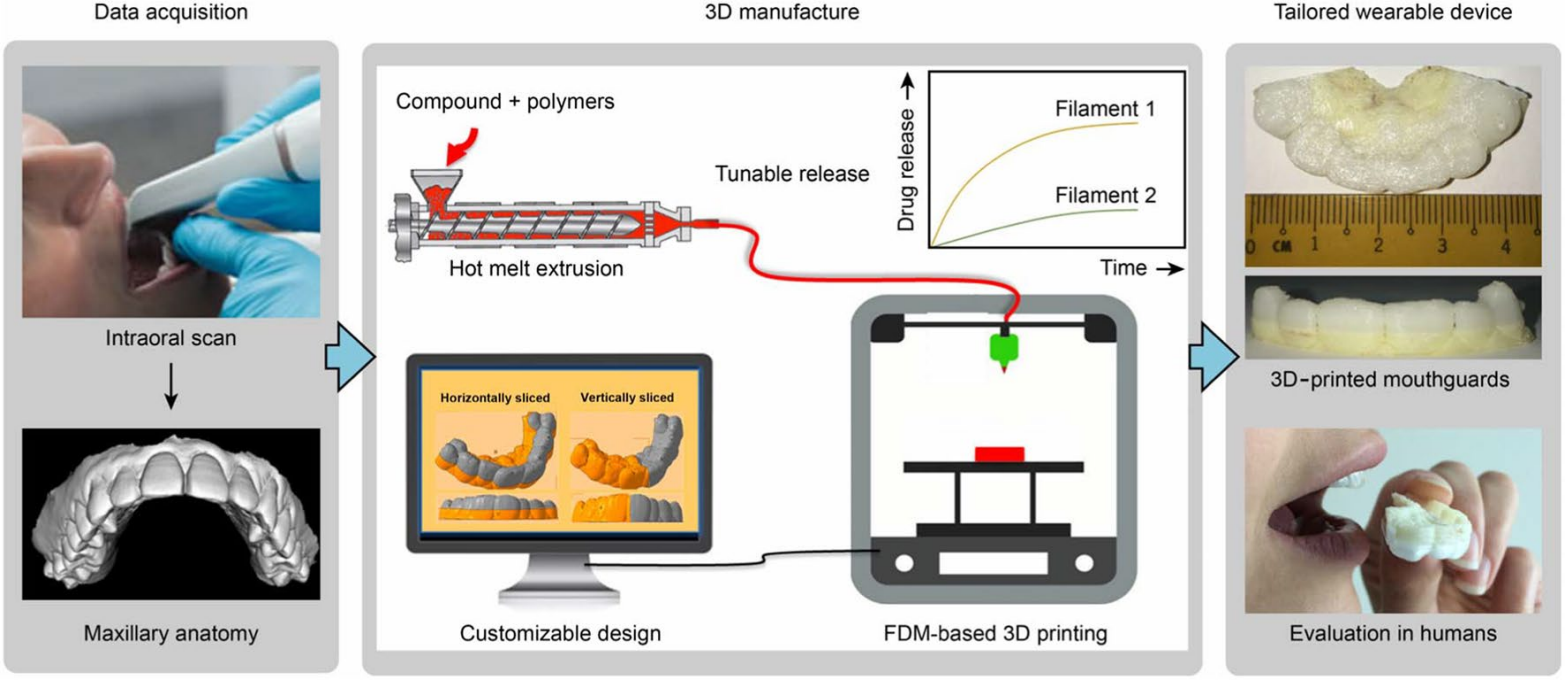
Workflow for the manufacture of wearable personalized oral delivery mouthguards by 3D printing [49]

Personalized 3D-printed mouthguards (Fig. 2), widely used devices for dental protection and alignment, are used not only to deliver drug substances in the oral cavity but also as diagnosing tool, e.g., to determine the lesion areas [12, 41, 49].

Liang et al. [49] used 3D printing by fused deposition modeling (FMD) for the manufacturing of wearable personalized oral delivery mouthguard. The filaments for manufacturing the device were prepared by hot melt extrusion (HME) technique using poly(L-lactic acid) (PLA) and poly(vinyl alcohol) (PVA) as thermoplastic polymers. The personalized prototype devices were designed based on scanned templates obtained with an intraoral scanner. In the study, involving six volunteers, it was found that salivating pattern did not play an important role in conducting the release of vanillic acid from the mouthguard, but wearing the mouthguards led to some minor discomfort for the volunteers due to imperfect fitting and hindrance of speech.

Hildebrandt et al. [37] evaluated mouthguards coated internally with an antiseptic chlorhexidine varnish. Twenty-two patients with salivary *Streptococcus mutans* level ≥ 10^4^ CFU/ml wore the device for an average of 7 h for 7 nights, and a significant reduction in salivary bacteria levels was observed in the samples immediately after treatment and 1 and 3 months later. Similarly satisfying results were observed in 46 pediatric patients wearing this device 1 week nightly [3]. Arnold et al. [7] showed that in another study, chlorhexidine was successfully delivered by the mouthguard device with a matrix composed of ethylene vinyl acetate matrix [7].

Personalized 3D-printed mouthguards allowed to achieve sustained release of fluoride, with a low burst effect [12]. The filaments for printing the mouthguard were composed of sodium fluoride and blends of poly(ε-caprolactone) (PCL) and poly(vinyl alcohol) (PVA) or poly(ethylene glycol) (PEG). *Ex vivo* studies were performed on decayed human teeth using the 3D-printed tooth caps that precisely fit the complex geometrics of each specimen. This study did not take into consideration, however, the complex mechanism of caries’ formation of the lesion and the constant renewal of saliva [12].

### Slow-release fluoride glass (SFG)

**Fig. 3:**
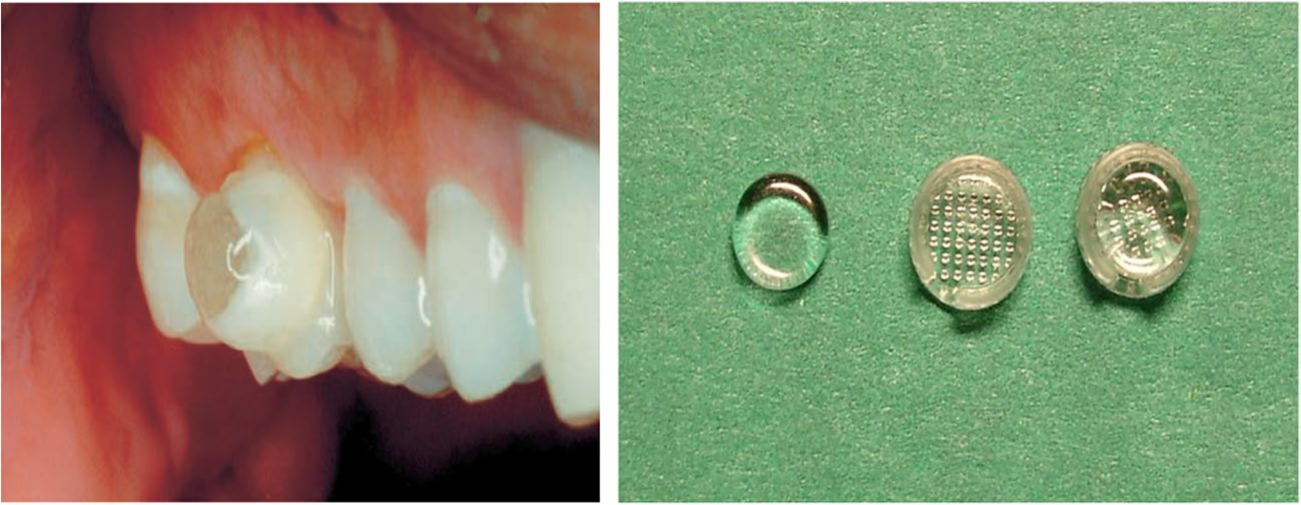
Fluoride slow-release glass device attached to the buccal surface of an upper first permanent molar tooth (left) [83]. The latest version of the fluoride glass slow-release device and plastic retention bracket (right) [82]

Many other studies were conducted to evaluate the release of fluoride from slow-release fluoride glass pellets (SFG) (Fig. 3, left side [83]). The glass device was developed by Curzon in 1984 [2]. It dissolves slowly when it is moist, thus releasing fluoride without affecting the device’s integrity sufficiently. The original device was dome shaped (4 mm in diameter) attached to the buccal surface of the first permanent molar using adhesive resins [84]. Due to the low retention rates of the original device, it was further substantially changed: the device has been shaped in the form of a disk that is placed within a plastic bracket to facilitate device handling, attachment, and replacement [65] (Fig. 3, right side [82]). The device was shown to be capable of maintaining low levels of fluoride in the saliva of ~ 0.1 ppm (i.e., up to 10 times that in normal control saliva) [1]. Some publications mentioned that the main problem related to these devices was the easy detachment [49]. Curzon and Toumba [20] showed that the prototype SFG was retained in the mouth and released fluoride into saliva; a mean concentration of 0.030 mg/l was achieved lasting for over 18 months.

### Intraoral fluoride-releasing system (IFRS)

**Fig. 4:**
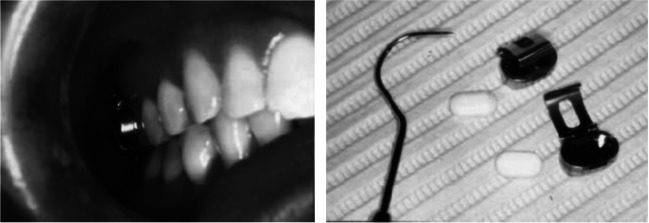
Intraoral fluoride-releasing system and fluoride pellets (right) and their appearance when placed on a maxillary molar (lift) [58]

The IFRS is a membrane-controlled reservoir-type device (half-football shaped) (Fig. 4 [58]) that consists of an inner core and an outing coating. The inner core contained sodium fluoride dispersed in an acrylate copolymer composed of hydroxyethyl methacrylate (HEMA) and methyl methacrylate (MMA). The core was coated with a HEMA/MMA film. Each fluoride pellet contains 35 mg of sodium fluoride and is designed to release a mean of 0.12 mg of fluoride per day for 130 days [18, 58]. The release of fluoride from the device is controlled by the degree of hydration and the thickness of the rate controlling membrane [17].

Meyerowitz et al. [58] studied the anticaries effectiveness of an IFRS with a standard regimen of daily application of a 1.1% neutral sodium fluoride gel in custom trays. The patients, with radiation-induced xerostomia, were satisfied with the device. The discomfort with the device was minimal and only some patients reported some difficulty in chewing with the device in place. A slight inflammation or ulceration on the buccal mucosa opposite the IFRS device was observed in 30.8% of the patients.

Chambers et al. [18] compared the treatment with either IFRS or stannous fluoride gel (control group)—22 head and neck cancer patients with radiation-induced xerostomia were entered in this study. The IFRS is designed to release a daily dose of 0.12 mg of sodium fluoride, with a single application of 4 months. It would be more convenient than the daily home application of a tray of fluoride gel and avoids the problem of variable patient compliance. The resting and stimulated salivary flow rates were significantly greater in the control group than in the study group.

### BuccalDose® system

**Fig. 5:**
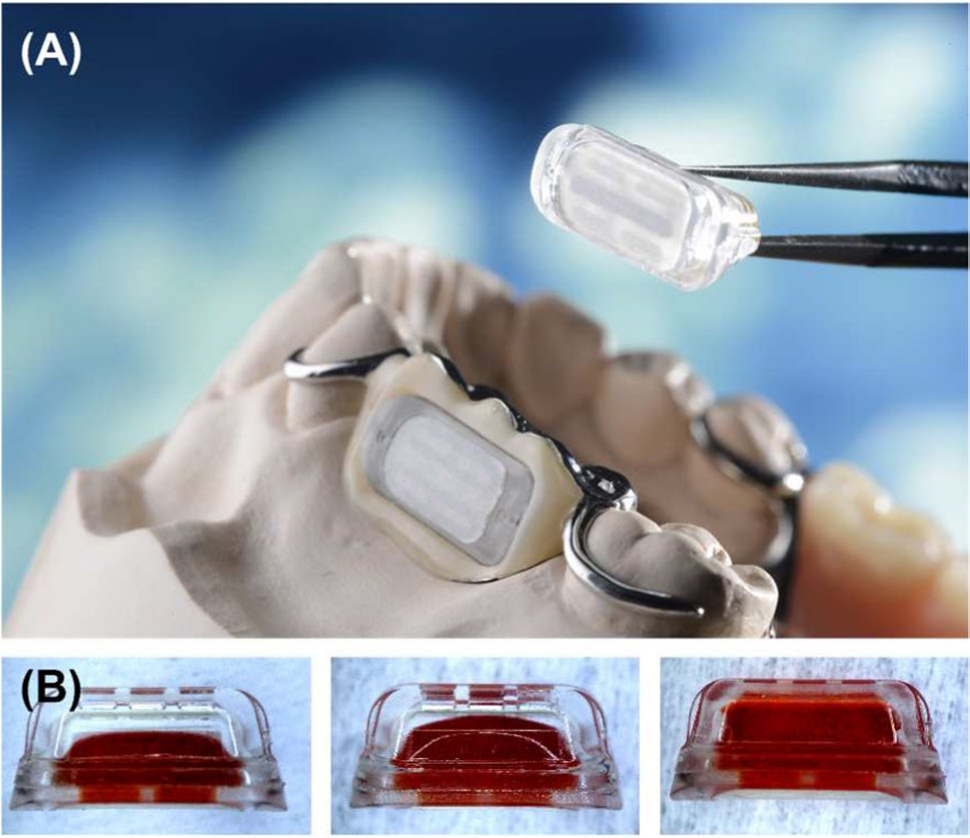
BuccalDose®—intraoral drug delivery system integrated in a model cast partial denture (**A**); the cartridges (**B**) [33]

BuccalDose® is a disposable intraoral drug delivery cartridge (Fig. 5 [33]) for the self-medicated treatment [79]. The system is intended for the treatment of Parkinson’s disease with dopamine agonists to match the needs of elderly and impaired people and currently is under pre-clinical development [34, 36]. The cartridge contains an osmotic pump and consists of the following components: (i) a microinjection molded housing made from a cyclic olefin copolymer (COC), (ii) a semi-permeable polyamide thin film composite membrane, (iii) a hyperelastic styrene copolymer (SEBS) barrier membrane separating the osmotic agent from the drug, and (iv) fluidic capillaries for drug release [35]. The disposable cartridge is magnetically attached to the receptacle of a partially removable prosthesis. By applying an assistive tool, the cartridge can be handled even by patients affected by motility disorders [36]. Using models of the jaw of patients with at least two missing teeth in the lower jaw, it was shown that in 183 out of 200 cases, an easy and comfortable installation of the device was possible, either in patient-owned or custom-made prosthesis [33].

The main problem related to this system was that the removal of the cartridge from the prosthesis with the assistive tool was difficult for patients with more limited motor skills in moderate or advanced disease stages. The degree of saliva secretion is a very important factor to obtain proper operation [34, 36].

### Microneedles

Microneedles (MNs) differ in length (25–2000 μm) and shape. They perforate stratum corneum or mucosal membrane in a safe and practically non-painful manner [23, 71]. The patches with MNs have actively been investigated as a technique of physical enhancement of transdermal drug delivery [5, 51, 71] but they offer potential advantages also for drug administration through mucosal membranes. MNs have already been investigated in the studies of oral mucosal vaccination, oral cancer management, and gastrointestinal drug delivery to enhance delivery across oral and gastrointestinal parries [71].

MNs are made of numerous materials (silicon, ceramics, glass, sugars, biodegradable polymers, steel, etc.) [71]. Different fabrication methods such as microfabrication, micromachining techniques, drawing lithography, and advanced molding technology have been used to make MNs with different shapes. Three-dimensional (3D) printing has recently been used to produce MNs as well [47, 48, 64].

**Fig. 6:**
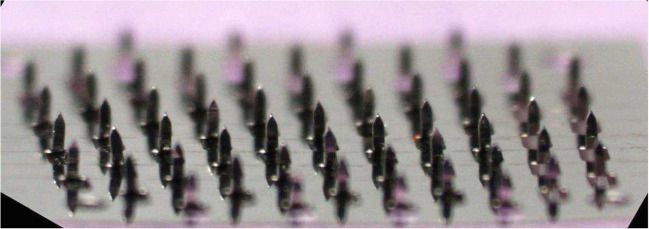
Drug-coated MN array for treating oral cavity cancers [54]

MNs were studied for the localized treatment of oral carcinoma, where conventional hypodermic injections have poor distribution and low retention while also causing pain to the patient [48]. Ma et al. [54] developed coated MNs as a device for the delivery of anticancer drug doxorubicin. Doxorubicin was encapsulated in poly(lactic-co-glycolic) acid (PLGA) nanoparticles (137 nm) which were coated on in-plane 1D microneedles (Fig. 6). Microscopic evaluation of 3D tissue phantoms and porcine cadaver buccal tissues demonstrated that doxorubicin could diffuse both laterally and vertically into the tissues and produced cellular cytotoxicity. Human insulin and human growth hormone were also successfully delivered to the buccal cavity of swine using a highly drug-loaded MN patch [14].

MNs coated with sulforhodamine, ovalbumin, and two HIV antigens were inserted into the inner lower lip and dorsal surface of the tongue of rabbits. Using ovalbumin as a model antigen, it was found that importantly, the lip and tongue induced a significant secretory IgA in saliva compared to pre-immune saliva. Microneedle-based oral cavity vaccination also significantly increased the levels of antigen-specific IgA in saliva compared to the intramuscular route using two HIV antigens [53].

A new method of vaccination to the buccal mucosa using microneedles and cholera toxin adjuvant was also studied using a buccal tissue in vitro [61]. Serpe et al. [77] used sulforhodamine as a model drug, and in vitro permeation experiments were conducted for simulated dynamic and static saliva flow (phosphate-buffered saline was used) in the donor compartment of the Franz diffusion cell, into which buccal tissue was placed. This study showed the importance of including salivary flow during in vitro studies related to the use of medical devices for drug delivery to the oral cavity to not obtain misleading results.

Caffarel-Salvador et al. [14] conducted a trial on 100 healthy volunteers to assess the patient’s comfort with a patch with MNs applied to the buccal cavity. The hard palate was reported to be the preferred site of the patch application.

### Iontophoresis

**Fig. 7:**
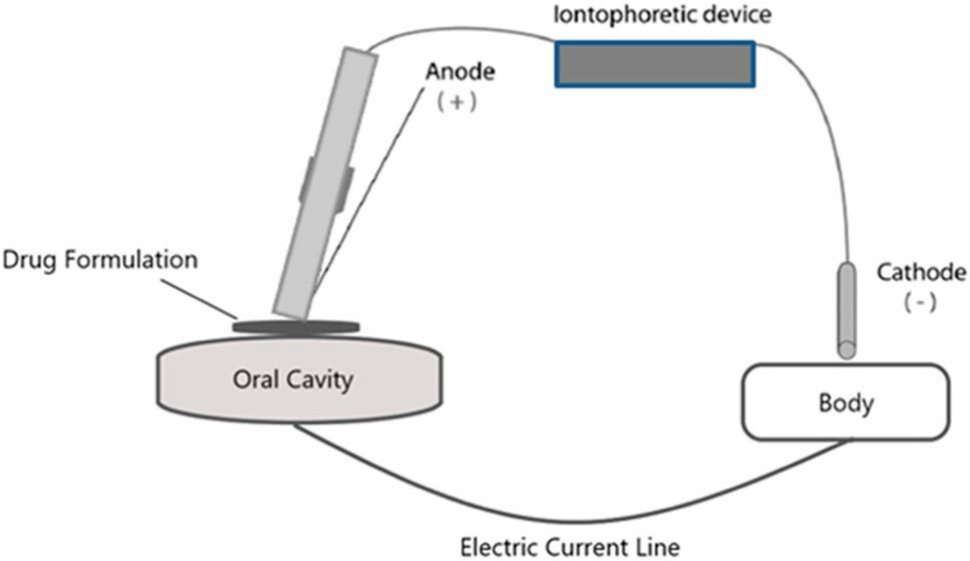
Schematic drawing of a principle of iontophoretic delivery of an active substance in the oral cavity [86]

Various physical techniques have been explored to achieve sustained drug delivery in the oral cavity including electroporation, sonophoresis, and iontophoresis [29, 69]. Iontophoresis enhances the delivery of both charged and uncharged drugs by using an electrical current (Fig. 7 [86]). The mechanisms of iontophoresis include electrophoresis, electroosmosis, and electropermeabilization (electroporation). The mucosa, palate, enamel, and dentin in the oral cavity are studied for the iontophoresis technique [57, 67, 86]. Some drugs, e.g., lignocaine or sodium fluoride, are already administered by iontophoresis to patients effectively. Others like dexamethasone, 5-fluorouracil, leucovorin, lidocaine, nicotine, ondansetron, metronidazole, acyclovir, amikacin, gentamicin sulfate, ciprofloxacin, amoxicillin, ketoconazole, fentanyl hydrochloride, diclofenac sodium, ibuprofen, naproxen sodium, and potassium iodide are under in vitro investigations or undergoing animal studies and have shown potential for applying them for delivery them [40, 42, 43, 67, 86, 87]. To facilitate the iontophoretic transport of ions into the enamel, two devices were developed: Fluorinex® (for sodium fluoride) and “electrically accelerated and enhanced remineralization” (EAER®) [30, 56, 66].

**Fig. 8:**
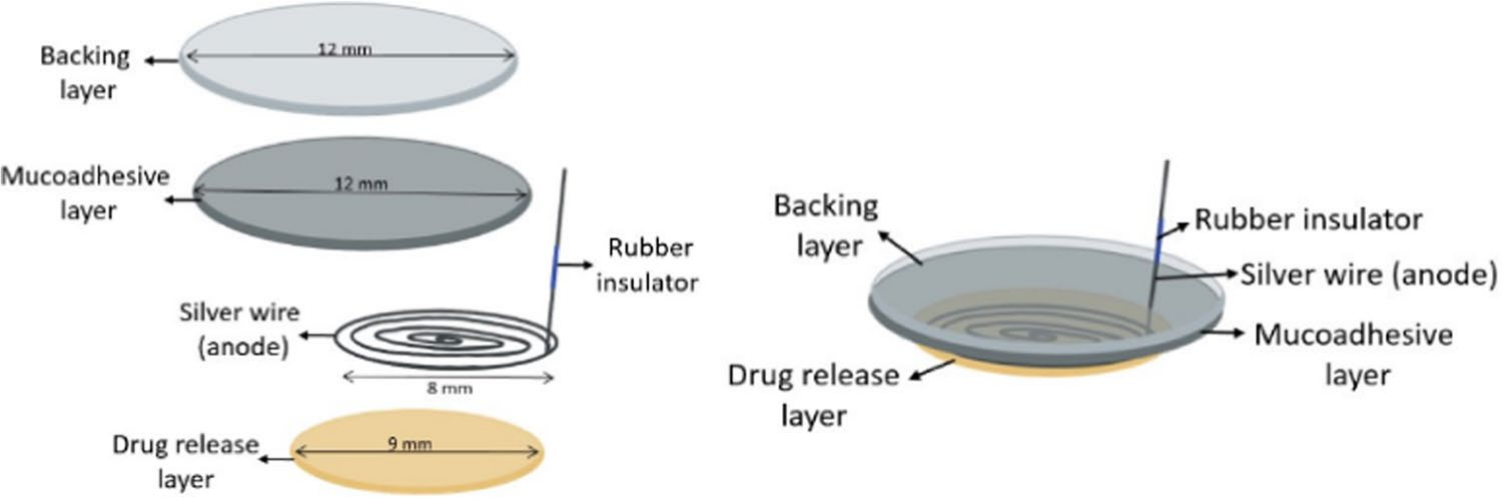
Graphical representation of buccal tri-layered iontophoretic patch [24]

**Fig. 9:**
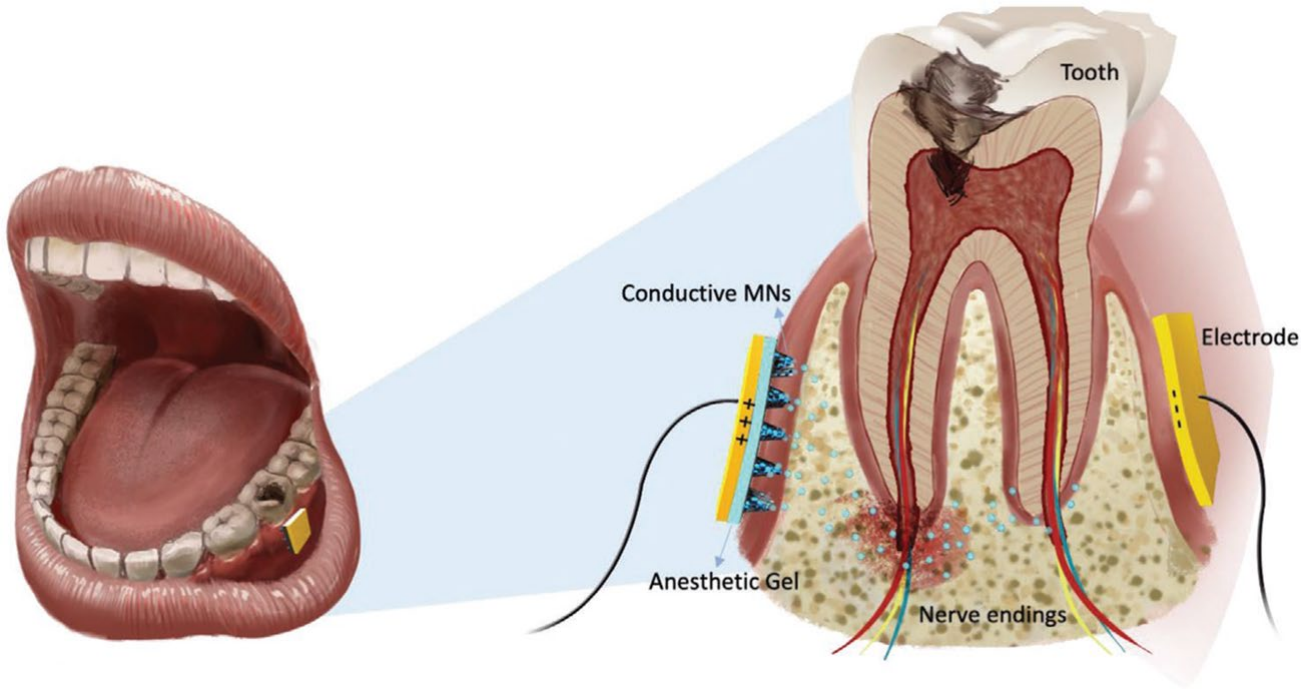
Schematic diagram demonstrating microneedles in combination with iontophoresis for directed delivery of anesthetic drugs to deep tissues in density [76]

The principle of operation of an iontophoretic device is to place the electrode on the gel/solution containing the active substance. So the device itself is not introduced to the oral cavity, like the devices described above. Therefore, only the systems that use electrical potential in combination with an adhesive system or another drug reservoir are characterized in this review. Besides IntelliDrug described below, two systems should be mentioned.

do Couto et al. [24] proposed a patch with drug delivery promoted by the current for anesthetic delivery in the buccal epithelium. The buccal patch was designed to be tri-layered: drug release, mucoadhesive, and backing (Fig. 8). An anode spiral electrode was placed in between the drug release and mucoadhesive layer to enable iontophoresis. A mixture of hypromellose and polyethylene glycol was used to prepare the drug release layer. In another system, the strategy of conductive microneedles array was employed. The MN patch was used in combination with the iontophoresis technique (Fig. 9) and delivery of lidocaine through oral mucosa succeeded in a clinically relevant rabbit incisor model [76].

#### IntelliDrug®

**Fig. 10:**
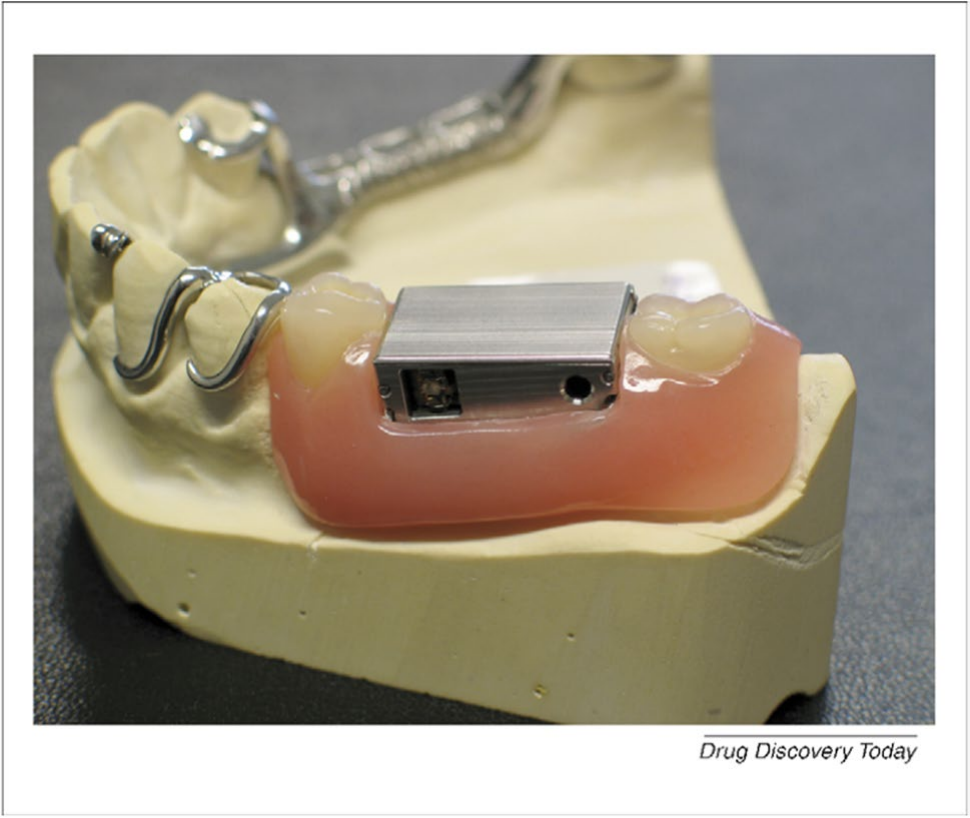
A non-miniaturized “IntelliDrug” prototype embedded in a partial lower jaw denture [73]

Among the applications of the iontophoresis technique is an IntelliDrug® device, a self-contained delivery device for the oral cavity that has been developed by a multinational EU consortium (FP6-IST project “IntelliDrug”). IntelliDrug® device is a single-component osmotic pump and integrated controlled drug delivery system to be implanted into the human denture, releasing the drug through oral mucosa [21, 31, 59]. The system (Fig. 10), which is the size of two molars, consists of a stainless steel intraoral module containing an osmotic membrane, a drug reservoir that could additionally contain chemical enhancer, an actuation mechanism to push the drug solution, a drug level sensor, a flow sensor, a power source, software, and outlet system with iontophoresis electrodes [38].

The safety and effectiveness of the device were studied on patients receiving naltrexone, widely used in the treatment of opiate addiction [16, 63]. The IntelliDrug® prototype functioned properly and it did not exert any side effects. In addition, the transbuccal route resulted in 4–17 times higher bioavailability of naltrexone than achieved by conventional *per os* route. Giannola et al. [28] conducted an in vivo study on pigs with galantamine intended to treat Alzheimer’s dementia and highlighted the advantage of this method of treatment compared to injectable formulations. Scaturro et al. [75] evaluated the effect of pH on the buccal delivery of levodopa methyl ester prodrug (levodopa, the most effective pharmacologic agent in Parkinson’s disease) in *ex vivo* permission behavior (to use it as a drug model in IntelliDrug® mechatronic device). However, further clinical studies are needed to confirm the use of this approach as a new effective tool for the maintenance of constant dopamine stimulation (the main goal in the treatment of Parkinson’s disease).

The IntelliDrug® system requires sufficient saliva secretion flow to work properly. Because of this, the system might be contraindicated in individuals suffering from xerostomia or dry mouth [36]. Another disadvantage of this device is that the patient needs to have two missing adjacent teeth [10].

## Conclusions

Intraoral systems may become an excellent approach to the local or systemic drug delivery. There is a growing interest in this approach for administration of medicines for chronic diseases such as Parkinson’s disease or Alzheimer’s disease. Although some of the devices achieved success through the experiments that were conducted in vitro or in animal models, more studies must be carried out in vivo to prove the feasibility and therapeutic efficacy of the proposed ideas. The main challenge is patient’s compliance and comfort when placing a foreign object inside the oral cavity. 3D printing plays an important role in the development of such devices through the possibility of fast manufacturing of personal devices that fit individual patients. On the other hand, the patches with microneedles can overcome the problem of poor absorption of many drug molecules, including biopharmaceutics and, in contrast to the iontophoretic devices, are more acceptable due to smaller size and comfort.
